# Clinical Considerations on Infectious Gingivites

**Published:** 1896-08

**Authors:** Charles Greene Cumston


### International Dental Journal.

Vol. XVII. August, 1896. No. 8.

### Original Communications.[1]

### CLINICAL CONSIDERATIONS ON INFECTIOUS GINGIVITES.[2]

BY CHARLES GREENE CUMSTON, B.M.S., M.D.[3]

Inflammation of the gums may have either an acute or chronic course, but no matter which this course may be, the affection will take on the three following stages,—viz., stage of simple infection, stage of suppurative infection, stage of destructive infection.

Each of these stages can manifest itself independently from the others, according to the cause of the infection or the character of the soil in which it is evolved. Consequently, the single types corresponding to the three following affections are to be met with in practice,—viz., *simple gingivitis*, *purulent gingivitis*, and, lastly, *gangrenous gingivitis.*

I will pass these three types rapidly in review. Simple gingivitis has also been described as erythematous or catarrhal. The beginning of the infection is occasionally marked by a general malaise, but usually of so slight a degree as not to be remarked by the patient. Anorexia may be pronounced, as well as a dryness of

---

[1] The editor and publishers are not responsible for the views of authors of papers published in this department, nor for any claim to novelty, or otherwise, that may be made by them. No papers will be received for this department that have appeared in any other journal published in the country.

[2] Read before the Harvard Odontological Society, October 31, 1895.

[3] Instructor in Clinical Gynæcology, Tufts College; member of the Société Française d'Electrothérapie; Director of the Department of Gynæcology, Tremont Dispensary.

the buccal cavity, the latter symptom calling the patient's attention to his month.

The mucous membrane is hot, red, and rather painful. Soon there is a difficulty in moving the jaws, the breath has a disagreeable odor, and salivation is increased.

If the mouth is examined, the gums will be found in a state of tumefaction and soft to the feel. A bright-red line, having a width of two or three millimetres, situated on the free border of the gums, is easily seen, as it contrasts markedly with the dark-red color of the remainder of the gums. Here and there a whitish film, covering the inflamed gums, will be observed, usually in the neighborhood of the teeth. Sometimes the glands will become indurated and enlarged.

This form of gingivitis is generally very easily and quickly cured; however, it can transform into the suppurative form, which I will now take up.

The suppurative gingivitis is characterized by superficial ulcerations, and is ushered in quite often by fever. The patient does not feel himself; he is tired, has loss of appetite, and complains of his mouth as being dry and painful.

Then movements of the jaws become painful, and even impossible, due to the ulcerations on the gums and the indurated lymphatic glands.

The pain becomes dull and steady, and the patient, who can neither eat, sleep, nor speak, becomes prostrated.

The physical signs are here more marked than in simple gingivitis. The transparent epithelium is raised up by a serous exudation, but which rapidly becomes purulent. The epidermis falls, leaving the swollen and thickened dermis bare. This surface bleeds easily, and is of a bright red color, unless, as it sometimes happens, it is covered by a kind of dirty membrane, in which alimentary *débris*, mixed with the products of buccal excretion and secretions, especially tartar, are to be found.

It is under this more or less thick layer that the superficial ulcerations form, and which, although they quite often remain circumscribed, may extend not only all over the gums, but to the internal aspect of the cheeks and lips as well. There is consequently great salivation and fetidity of the breath ; the submaxillary glands become tumefied and most painful.

In eight or ten days this suppurative form will get well, under proper treatment; but if not, it passes into the chronic state, or becomes gangrenous.

Gangrenous gingivitis may succeed the preceding forms, but more often it appears at the same time in patients recovering from some debilitating general malady. Patients feel sick, and complain of cephalalgia, anorexia, and vertigo.

Hemorrhages from the gums make their appearance; the great quantity of saliva is most troublesome to the patient, who finds that his gums are painful, swollen, soft, and granular, while around the teeth notches will be felt. This is the first or fungous stage.

A whitish-gray deposit, a sort of pseudo-membrane, soon forms on the free borders of the gums, and underneath this are to be found ulcerations, which extend in size and depth, producing repeated hemorrhages and undermining the teeth, causing these to fall out. This is the stage of ulceration.

The infectious process continues its work. The mucous membrane, which was dark red, becomes earthy in look; blackish patches of necrosis become detached, leaving the alveola bare. This ravage may continue in every direction to such an extent that death is the result.

The functional symptoms undergo the same increase; anorexia and insomnia are complete; the patient can neither eat nor speak, so great are his sufferings. Diarrhœa appears, the pulse is small and frequent, and a dyspnœa shortly precedes the comatose state, in which the patient dies.

A quick and proper treatment will be able to save patients with gangrenous gingivitis, but a cure is infrequent when once the symptoms of auto-intoxication have appeared.

There are intermediate forms of this affection,—for example, vegetating fungous gingivitis and hypertrophic gingivits,—but these are chronic types, and have little tendency to progression.

I now come to the study of infectious gingivitis, properly speaking, and shall purposely omit the toxic and trophic forms.

*Scarlet Fever.*—An inflammation of the gums may occur between the tenth and the fifteenth day of scarlet fever. This is an infrequent complication; it may come on insidiously, but it may also be ushered in by severe general symptoms resembling a typhoid condition. The patient rapidly loses flesh, vomits continually, and an obstinate diarrhœa is present. The urine will be found to contain albumen. The pulse is small and rapid. Dyspnœa and delirium may supervene, and death is the result.

Scarlatinous gingivitis is quickly over in slight cases, and is characterized by tumefaction and redness of the mucous membrane, which readily bleeds.

The most frequent form in this disease is the ulcero-membranous gingivitis, which may become gangrenous. Pain is most acute, preventing movements of the jaw; the saliva runs away from the mouth, and the breath is very fetid. Yellow pseudo-membranes appear on the free border of the gums, covering the ulcerations, which may extend in surface and depth, producing necrosis of parts of the mucous membrane. The infection may invade the lips and internal aspect of the cheeks; the submaxillary glands are greatly enlarged.

The prognosis depends on the gravity of the lesions. Good when they are superficial, it becomes very bad when the infection becomes general. The alveolo-dental ligament may be invaded and destroyed, and the infectious process may even attack the alveolæ. Excepting in the last-mentioned cases, where the affection may be the source of secondary infections and of severe local lesions, demanding a considerable period for their cure, the gingivitis of measles disappears quickly.

*Aphtha.*—Chills, slight fever, headache, insomnia, and painful gums mark the commencement of aphthous gingivitis. The mucous membrane is red, bleeding, very painful, and tumefied. Soon the surface, especially around the implantation of the teeth, is covered by an eruption of confluent white vesicles, giving rise to superficial ulcerations. From the gums these vesicles invade the entire buccal mucous membrane. Sometimes the vesicles disappear, and there remain large patches of necrosed mucous membrane near the last molars, resembling ulcero-membranous stomatitis, while the borders of the gums are in a fungous and suppurating condition. In such cases the patient presents a typical typhoid condition, with gastro-intestinal symptoms, vomiting, and diarrhœa, while albuminuria, nervous symptoms, and adynamia are present. This gingivitis is most painful, and mastication is rendered still more difficult by enlarged glands, which may be considerable.

The aphthæ may be absolutely wanting; neither herpes nor ulcero-membranous stomatitis are present, and nothing but a pearly spider's-web membrane covers the gums. (Pètre.) All lesions disappear in from a few days to six weeks by antiseptic treatment.

Scorbutus is too well known for me to discuss it, and will only be mentioned here.

*Gonorrhœal gingivitis* appears any time during the evolution of specific urethritis; however, according to Ménard, it is towards the end of the malady, and nearly always following other infectious complications that it is present. Thus arthritis, orchitis, and erythema precede the buccal symptoms of gonorrhœa. Then a great heat is felt in the mouth, with a continual thirstiness; the gums, especially the lower, are red, livid, tumefied, and bleeding, while the mucous membrane swells up between the dental arches.

Mastication and speech become difficult and painful; fever is quite high, and salivation and fetid breath render the patient's condition most disagreeable.

As the disease progresses a pseudo-membranous exudation covers the border of the gums, hiding the more or less deep ulcerations. The ulcerations are sharply cut and of a violet color. The internal aspect of the corresponding cheek presents at the same time, or successively, inflammatory lesions of the same nature. The lymphatics are enlarged.

Recovery from this gingivitis, which is not a contagion, but a *gonorrhœal infection*, is complete in about two or three weeks. The treatment consists in cauterizations and antiseptic irrigations.

I now come to the pathological anatomy of the gingivites, which is certainly too long to be discussed in full, but I will endeavor to give a concise description.

Redness of the gums indicates the commencement of inflammation; it may be bright or dark; there is tension and swelling of the mucous membrane. The epithelium falls, leaving the dermis bleeding and uncovered. The blood and lymphatic vessels are dilated, and a serous exudation quickly appears, soon becoming purulent. The pus-cells may appear at the surface of the mucous membrane before the epithelium has been cast off, and are sometimes seen between the tumefied cells, thus contributing to their necrosis and desquamation. The epithelium is not always washed away by the saliva, but accumulates in the smallest crevices, especially in the *culs-de-sac* formed by the tartar between the tooth and gum, and when lodged there contribute to the formation of ulcerations and fissures.

The mucous membrane often becomes fungous, and also—as, for example, in scorbutus—may be covered with vascular granulations. Usually it is covered by a pultaceous membrane, which conceals the ulcerations underneath.

The ulcerations vary in number, extent, and depth; they may remain for a long time superficial, as in tuberculosis, or rapidly invade the underlying tissues. They may be preceded by maculæ, papulæ, vesicles, or pustules, as in scarlet fever, measles, etc., while in other cases they are covered by pseudo-membranes, as in diphtheria.

The bacteria, which abound, act not only by their infectious and toxic properties, but by their agglomeration ; they may prevent circulation by mechanical action. The blood-supply being thus cut off, the parts undergo necrosis, ulcerations form, and gangrene occurs.

The subdermic cellular tissue is generally in a state of œdema from the beginning of the gingivitis, while later in the progress of the affection it may be invaded by the infection and suppurate.

In some forms of the disease the secretions of the buccal cavity become very profuse, and even muco-purulent. This is due to an exaggeration of the activity of the glands of the mucous membrane, to which is added a sero-mucous exudation containing epithelial cells and leucocytes.

The consequences of a gingivitis are local and general. One of the most frequent local consequences is the generalization of the malady over the entire mucous membrane of the mouth. Diphtheritic gingivitis may be given as a type.

Gingivitis may cause the loosening and falling out of the teeth, due either to the propagation of the inflammation to the alveolo-dental ligament, resulting in its destruction, as in diabetes, etc., or, as is pointed out by Cruet, the alveolæ are first resorbed, and infection follows. If the infectious process continues, we have an acute osteitis of the jaw, its progress being rather rapid, with periods of remission and exacerbation. Suppuration occurs, forming sequestra, fistulæ, etc.

As the mucous membrane of the buccal cavity is very rich in lymphatics, it is easily understood how the submaxillary glands become involved. The adenitis may disappear with the gingivitis, but it can take on an acute type, in which case the periglandular cellular tissue becomes involved in the process. This periglandular inflammation may undergo resolution or suppurate.

Diffused phlegmon is very infrequent, and occurs in debilitated subjects. The prognosis is very bad, as death may occur from septicæmia.

Gingivitis renders alimentation difficult or impossible, and may in certain affections, such as tuberculosis, bring about death by cachexia. Salivation is also depressing, and does much to weaken the patient.

But the most serious consequence of gingivitis is *auto-intoxication.* This condition shows itself sometimes by gastro-intestinal troubles, vomiting, and diarrhœa. It may become so serious as to affect a typhoid type. In other cases the nervous system appears to be especially acted on by the toxines. Godelier and Barth each report a case of gingivitis followed by adynamia and death.

The infection may attack some particular organ, producing serious lesions, as, for example, Brissaud's case, in which an infectious gingivitis was followed by endocarditis and broncho-pneumonia, with death to the patient.

The diagnosis of the infectious gingivitis will be passed over, as this paper is already too long, and I will close by making a few remarks on the prognosis and treatment.

The prognosis is generally a question of *soil.* No matter what the local factor of the inflammation of the mucous membrane may be, if the *soil* is neither infected nor intoxicated, nor debilitated in any way, the lesions will be slight and recovery rapid. On the contrary, if the local infection finds a soil profoundly impregnated by toxines or weakened by a diathesis, its progress will be rapid and produce most serious lesions, as I have pointed out. Consequently it is not on the advanced stage of the buccal lesions, but on the general condition of the patient, on which the prognosis should be based.

As to the treatment. This should be directed to two factors,—viz., the general condition and the local lesions. I cannot here mention the treatment of each infectious disease producing a gingivitis, but will simply speak of the local care.

No matter what variety the gingivitis may be, it is a microbic manifestation, and in order to cure it the bacteria must be attacked; this is accomplished by severe antisepsis of the buccal cavity. First of all, the tartar should be removed, as it is a real culture medium, and a gargle of carbolic acid, one in two hundred; thymol, one in one hundred; or a more elegant and agreeable preparation may be chosen, such as Metcalf's spray solution, or listerine.

The permanganate of potassium I can highly recommend for buccal antisepsis, and I prescribe,—

℞ Kalii permanganat., 0.50;
Aq. menth. pip., 40.—M.
Sig.—From eight to ten drops in a glass of tepid water as a mouth-wash.

The use of saccharin combined with salicylic acid has also a favorable action in these cases, the following formula being an excellent combination:

℞ Saccharin,
Natrii bicarb., āā 1;
Acid. salicylat., 4;
Alcoholis, 150;
Aq. menth. pip., 50.—M.
Sig.—Half a teaspoonful to a glass of tepid water as a mouth-wash.

For the fungosities cauterization by chromic acid crystals or nitrate of silver, applied on cotton tampons, should be practised. In some cases the galvano-cautery may be necessary. Chromic acid is, I think, perhaps the better, as its action is limited to the point touched, and the slough is eliminated in three or four days. The epithelium will have appeared in a week or so after.

When there is gangrene the foci should be destroyed by the galvano- or the thermo-cautery.

Such is the local treatment of the infectious gingivites.

